# Author Correction: Understanding electrochemical switchability of perovskite-type exsolution catalysts

**DOI:** 10.1038/s41467-021-25320-0

**Published:** 2021-08-16

**Authors:** Alexander K. Opitz, Andreas Nenning, Vedran Vonk, Sergey Volkov, Florian Bertram, Harald Summerer, Sabine Schwarz, Andreas Steiger-Thirsfeld, Johannes Bernardi, Andreas Stierle, Jürgen Fleig

**Affiliations:** 1 TU Wien, Institute of Chemical Technologies and Analytics, Getreidemarkt 9/164-EC, 1060 Vienna, Austria; 2 Deutsches Elektronen-Synchrotron (DESY), 22607 Hamburg, Germany; 3 TU Wien, Institute of Materials Chemistry, Getreidemarkt 9/165-PC, 1060 Vienna, Austria; 4 TU Wien, University Service Centre for Transmission Electron Microscopy (USTEM), Wiedner Hauptstraße 8-10, 1040 Vienna, Austria

**Keywords:** Catalytic mechanisms, Electrocatalysis, Electrocatalysis, Nanoparticles

Correction to: *Nature Communications*, 10.1038/s41467-020-18563-w, published online 23 September 2020.

The original version of this Article contained an error in the Acknowledgements section, which was previously incorrectly given as ‘The authors gratefully acknowledge funding by the Austrian Science Fund (FWF) through project P4509-N16 as well as DESY (Hamburg, Germany), a member of the Helmholtz Association HGF, for the allocation of beamtime and provision of experimental facilities.’. The correct version states ‘The authors gratefully acknowledge funding by the Austrian Science Fund (FWF) through project F4509-N16 as well as DESY (Hamburg, Germany), a member of the Helmholtz Association HGF, for the allocation of beamtime and provision of experimental facilities.’ in place of the incorrect text. This has been corrected in both the PDF and HTML versions of the Article.

