# Supplementary material for: Understanding electrochemical switchability of perovskite-type exsolution catalysts
Source: Nat Commun. 2020 Sep 23;11:4801. doi: 10.1038/s41467-020-18563-w (PMC7511332; doi:10.1038/s41467-020-18563-w)
Supplement: Supplementary file 1 — Supplementary Information [file 41467_2020_18563_MOESM1_ESM.pdf]

Supplementary Information

to

Understanding electrochemical switchability of perovskite-type exsolution catalysts

Alexander K. Opitz[1]*, Andreas Nenning[1], Vedran Vonk[2], Sergey Volkov[2], Florian Bertram[2], Harald Summerer[1,3], Sabine Schwarz[4], Andreas Steiger-Thirsfeld[4], Johannes Bernardi[4], Andreas Stierle[2], Jürgen Fleig[1]

1) TU Wien, Institute of Chemical Technologies and Analytics, Getreidemarkt 9/164-EC, 1060 Vienna, Austria

2) Deutsches Elektronen-Synchrotron (DESY), 22607 Hamburg, Germany

3) TU Wien, Institute of Materials Chemistry, Getreidemarkt 9/165-PC, 1060 Vienna, Austria

4) TU Wien, University Service Center for Transmission Electron Microscopy (USTEM), Wiedner Hauptstraße 8-10, 1040 Vienna, Austria

*) *alexander.opitz@tuwien.ac.at*

Supplementary Figures

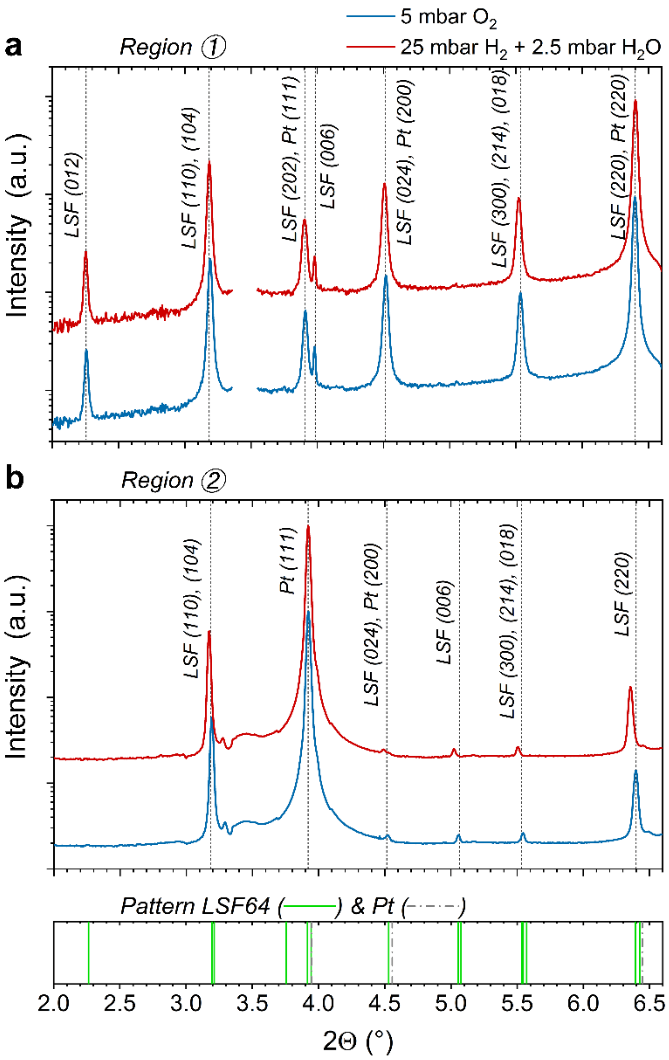

Supplementary Figure 1: Detailed phase analysis and anisotropic chemical expansion of $La_{0.6}Sr_{0.4}FeO_{3-\delta}$ thin films: Conventional 1D diffractograms were generated from the 2D diffraction patterns by integration; the corresponding integration regions are indicated in Figure 2 and supplementary Figure 2 by the numbered red "triangles". To point out the effect of the anisotropic chemical expansion, two integration regions at angles of $\chi \approx 0 - 5$ ° (region ①) and $\chi \approx 82.5 - 87.5$ ° (region ②) are depicted in (a) and (b), respectively. At 625 °C in 5 % O_2 only reflexes from rhombohedral perovskite-type $La_{0.6}Sr_{0.4}FeO_{3-\delta}$ (pdf # 00-049-00285) and face-centered cubic (fcc) Pt (pdf # 00-004-0802) can be identified, which patterns are both shown in (c).[1] The $La_{0.6}Sr_{0.4}FeO_{3-\delta}$ peak positions under oxidizing conditions are also indicated by the dashed gray lines in the background of the pattern. Immediately after switching the atmosphere to humidified hydrogen (H_2:H_2O = 10:1) a significantly different behavior is found for different χ angles. At very low χ virtually no peak shift can be observed, while at χ close to 90 ° a shift of the $La_{0.6}Sr_{0.4}FeO_{3-\delta}$ -related peaks to lower diffraction angles is evident. The corresponding change in the out-of-plane lattice constant amounts to 0.69 ± 0.04 %. This indicates that the chemical expansion, which is caused by defect chemical changes in the material,[2] does not occur isotropically in the entire film but only in the direction perpendicular to the film plane. This is due to $La_{0.6}Sr_{0.4}FeO_{3-\delta}$ being clamped in-plane by the substrate, while expansion is not hindered in the out-of-plane direction. The mechanical strain associated with this anisotropy may also change the defect chemistry of the perovskite oxide[3] and could thus be a reason for deviations of the chemical capacitance of $La_{0.6}Sr_{0.4}FeO_{3-\delta}$ thin film electrodes from the behavior of bulk-like samples.[4]

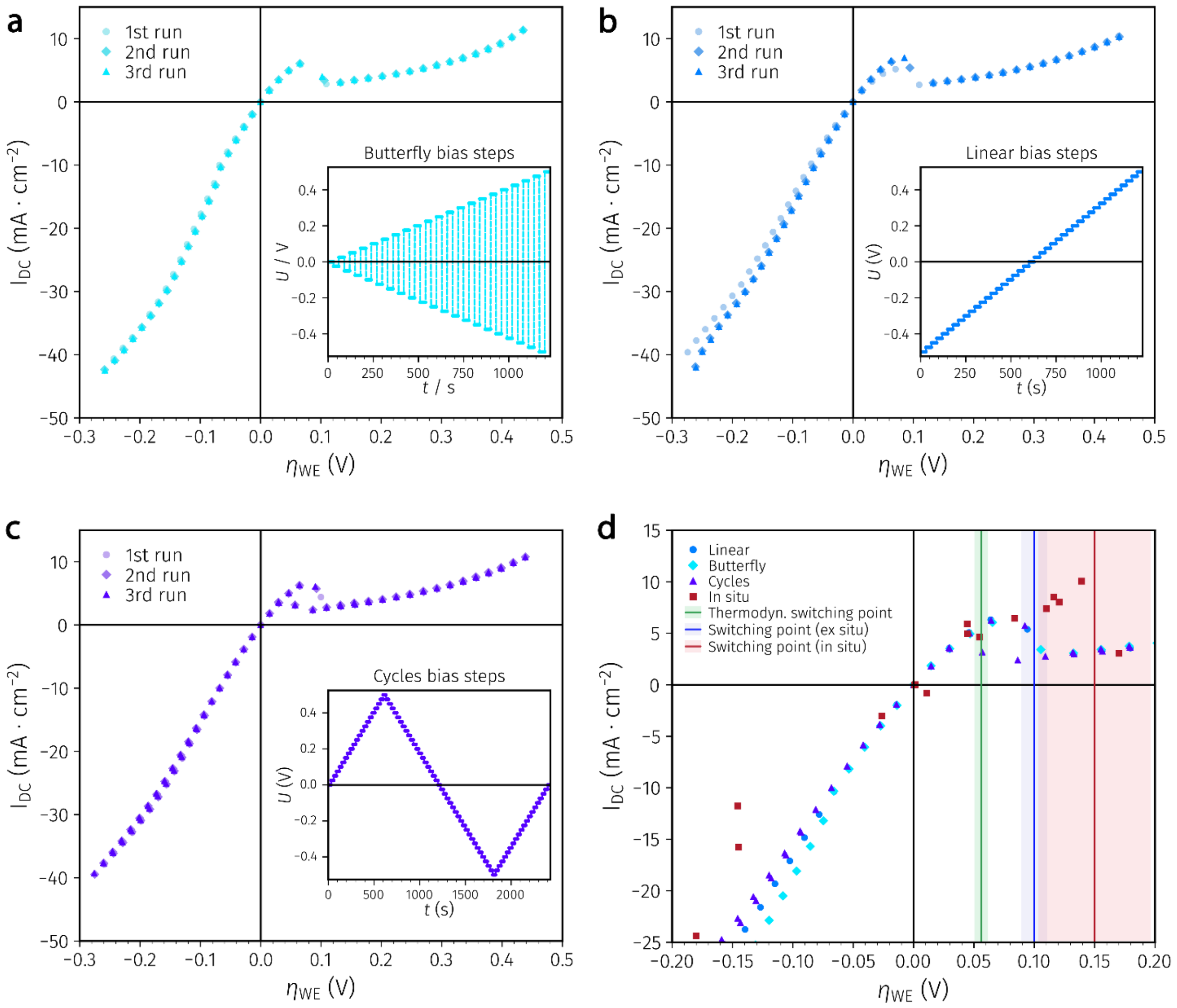

Supplementary Figure 2: Current density *versus* overpotential curves for $La_{0.6}Sr_{0.4}FeO_{3-\delta}$ with already existing surface decoration by exsolved iron particles. Depending on the voltage program applied – see insets – a slightly different switching behavior was found. (a) “Butterfly” program and (b) “Linear” sweeps result in a switching step. (c) In a “cycles” measurement a hysteresis-like behavior is found. Please note that (a) and (c) show the full measured range of Figure 7. (d) Comparison of 2nd run curves of each three measurement modes together with data points of Figure 3. The vertical bars indicate the Fe/FeO switching point obtained from thermodynamic data (56 ± 5 mV), from the current-overpotential-curve measured simultaneously to diffraction experiments (150 ± 46 mV), and from the current-overpotential-curves in (a)-(c): 100 ± 10 mV.

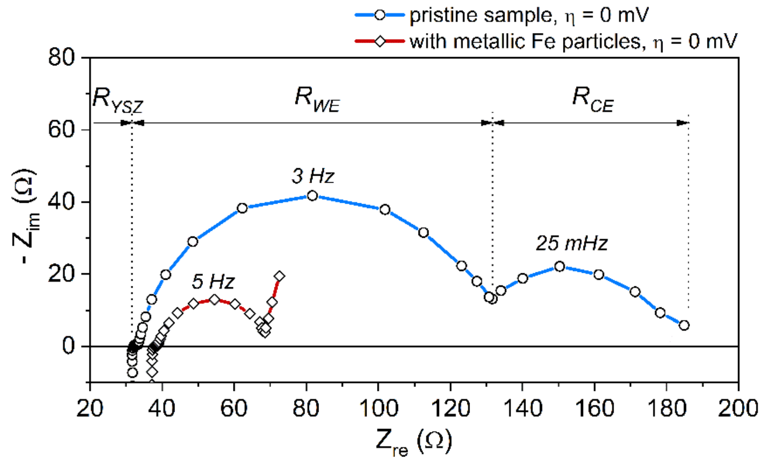

Supplementary Figure 3: Electrochemical impedance spectra measured at 625 °C in H_2:H_2O = 10:1 atmosphere without any bias voltage (η = 0 mV) on a pristine sample (i.e. before applying a cathodic bias; circles) as well as on a sample after a cathodic polarization, which triggered iron exsolution (diamonds). Both impedance spectra consist of the same features: The high frequency x-axis intercept can be mainly attributed to the resistance of ion conduction in the electrolyte; minor contributions are from wiring, contact resistances and electronic resistance in the $La_{0.6}Sr_{0.4}FeO_{3-\delta}$ film.[5] The slightly different high frequency intercepts for both spectra can be explained by small temperature variations during the experiment and by possible changes of the electronic conductivity of $La_{0.6}Sr_{0.4}FeO_{3-\delta}$ upon Fe exsolution. These two effects and possibly different contact resistances in case of changing samples are the origin for the uncertainty of the experimental temperature of 625 ± 10 °C. The intermediate frequency semicircle and the low frequency arc originate from the electrodes.[6] The larger (chemical) capacitance identifies the low frequency feature as the contribution from the porous $La_{0.6}Sr_{0.4}FeO_{3-\delta}$ counter electrode, while the medium frequency arc represents the thin film working electrode. This separation of resistive contributions from working electrode, counter electroce, and electrolyte is indicated by the arrows for the pristine sample. Please note that the appearance of a counter electrode contribution is most likely due to a gas diffusion limitation caused by the required way of mounting the samples. This increases the error bars of the overpotential at the working electrode. Applying a cathodic polarization to the thin film working electrode triggered exsolution of metallic Fe^0 particles and caused a strong change of the impedance spectrum at open circuit voltage. The corresponding working electrode polarization resistance without bias voltage becomes much smaller than for the pristine sample. This decrease of the working electrode polarization resistance – i.e. of the medium frequency semicircle – directly reflects the electro-catalytic activity increase of the $La_{0.6}Sr_{0.4}FeO_{3-\delta}$ electrodes upon Fe^0 exsolution. In principle, metallic Fe^0 particles are thermodynamically stable in the chosen atmosphere without applied bias. However, the very first exsolution of Fe^0 particles from the perovskite appears to be hampered and thus the pristine samples allowed the characterization of the electrochemical activity of the bare $La_{0.6}Sr_{0.4}FeO_{3-\delta}$ surface in reducing atmosphere.

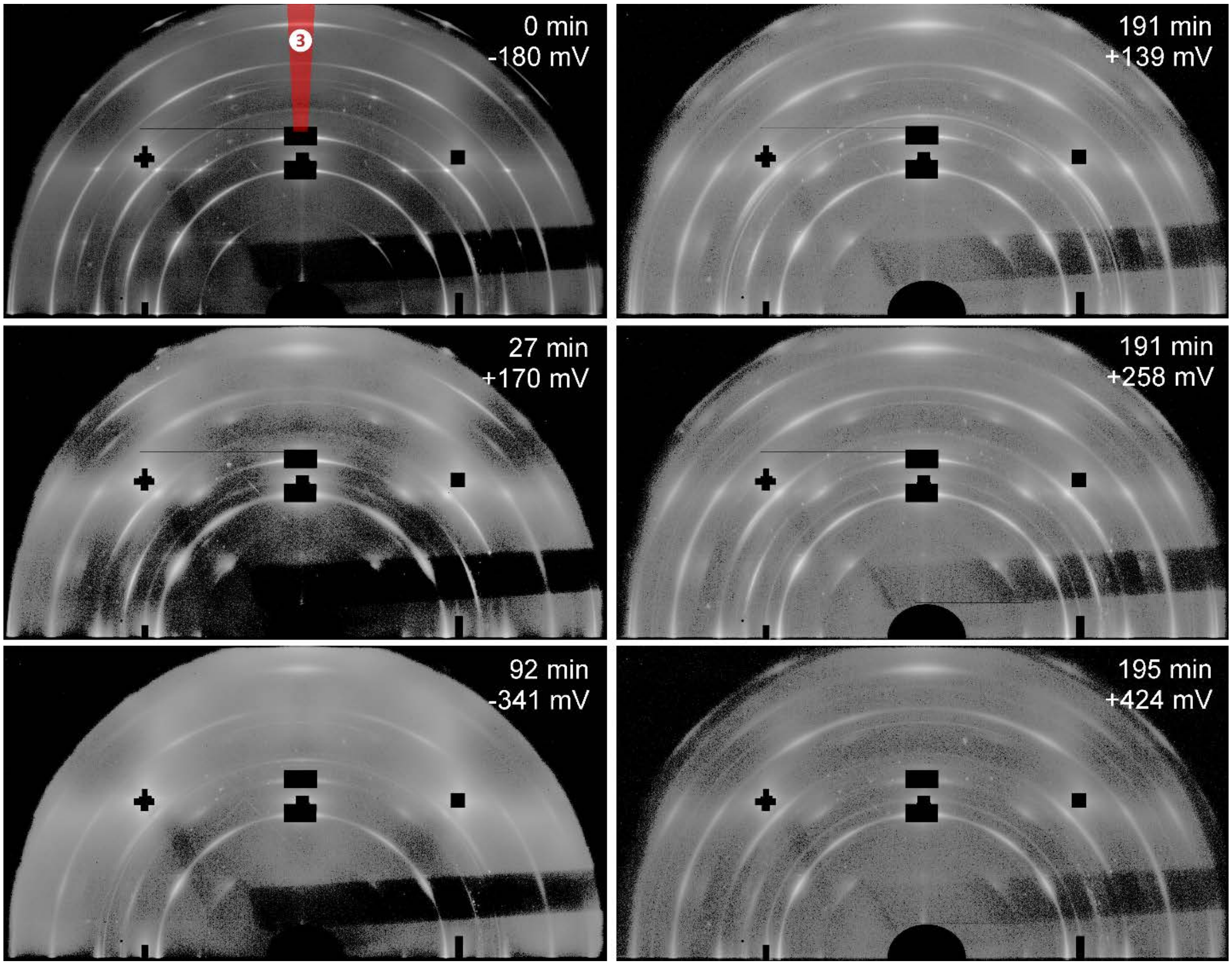

Supplementary Figure 4: 2D X-ray diffraction patterns measured *in-situ* on sample 2 under electrochemical polarization. The given time and overpotential correspond to different points in the currentcurve of Figure 3. Comparing the 2D diffraction patterns already indicates significant differences in the phase composition of the electrode, which can be seen by the appearance of additional Debye-Scherrer rings. For easier comparison and phase identification, conventional 1D pattern were generated from the 2D data by integration – see Figures 4 and 5 in the main text. The red segment in the top left image (at $\chi \approx 87.5$ -92.5 °) indicates the integration region for obtaining the 1D pattern in Figure 4.

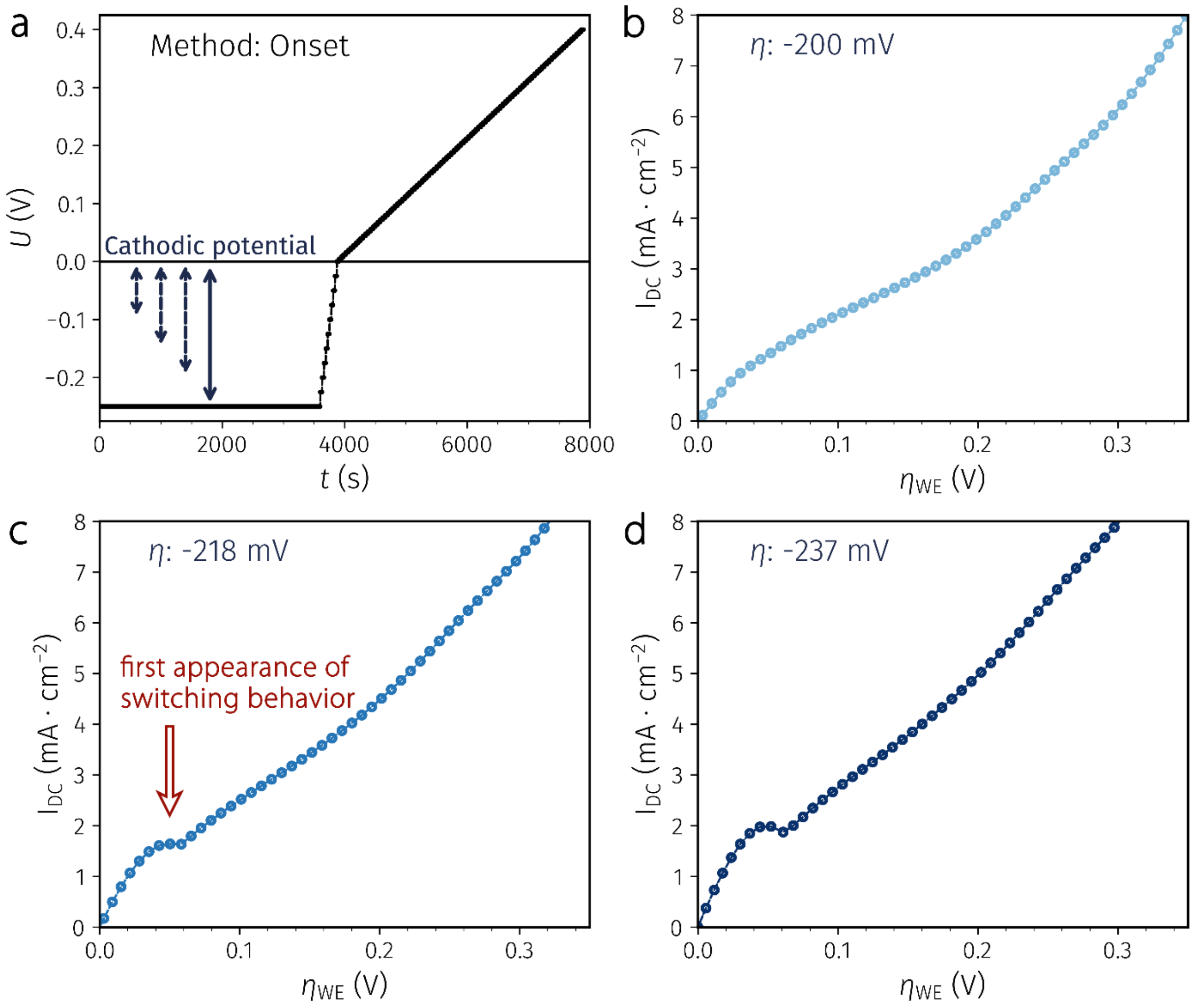

Supplementary Figure 5: Identification of the initial exsolution point. (a) Sketch of the measurement program used to capture the initial exsolution event. In (b), (c), and (d) the anodic branch of the current-overpotential-curve is shown after 1 h of cathodic polarization at -210 mV, -218 mV, and -237 mV, respectively.

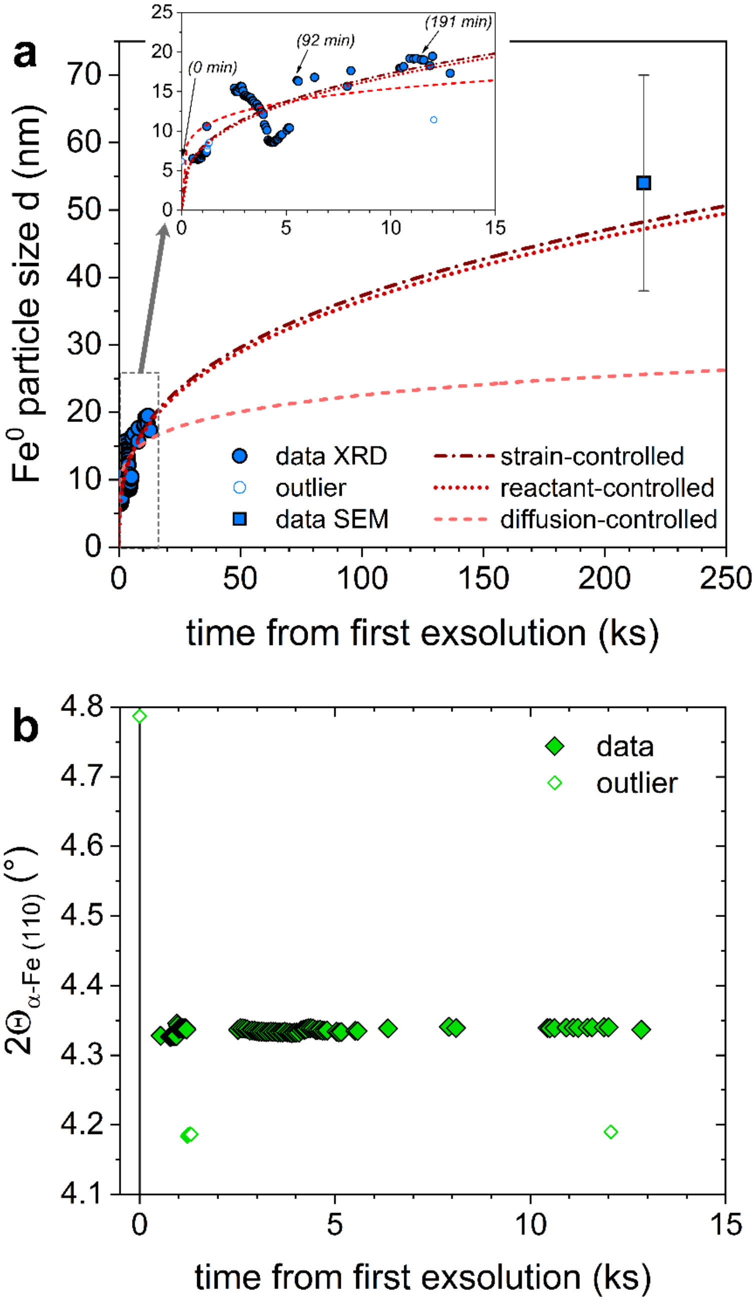

Supplementary Figure 6: Analysis of Fe^0 particle growth. (a) Evolution of the Fe^0 particle size with time. The starting time (t = 0) is the point of first observation of Fe^0 exsolution. The particle size was obtained from the width of the α-Fe (110) peak using equation 12 (circles, errors smaller than symbols) and from the electron microscopy image in Figure 6a (square; average ± standard deviation). The three lines are fit curves employing three different growth models (equations 13-15). The inset shows a zoom of the first 15 000 s (grey dashed box). (b) Diffraction angle of the α-Fe (110) reflex as a function of time from first exsolution.

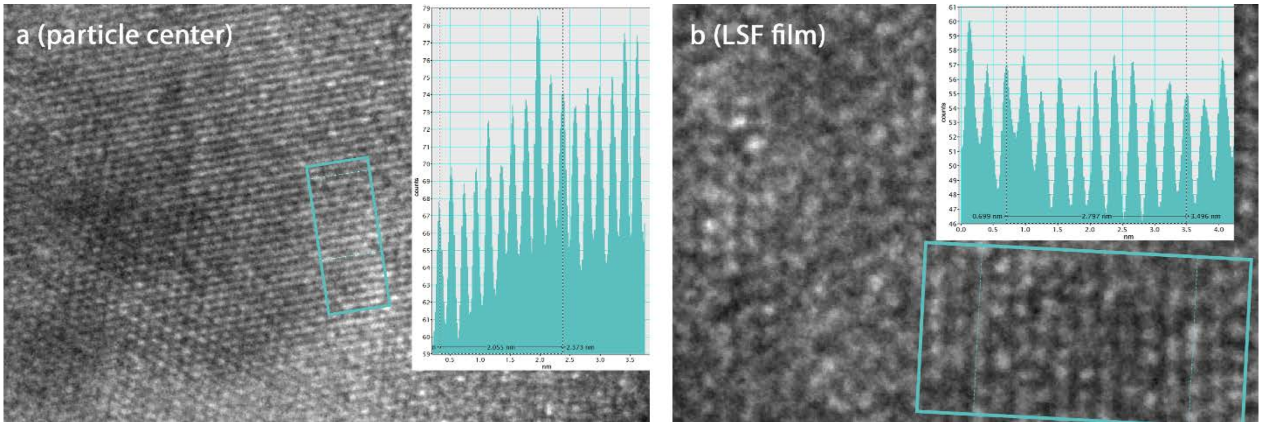

Supplementary Figure 7: Lattice plane spacings of the particle and the $La_{0.6}Sr_{0.4}FeO_{3-\delta}$ film. High resolution TEM images were recorded at (a) the center of the particle in Figure 6c and (b) on the $La_{0.6}Sr_{0.4}FeO_{3-\delta}$ film. From these images the lattice plane spacings were measured – see intensity *versus* position plots, which are shown as insets. Those were generated from the regions marked by the boxes.

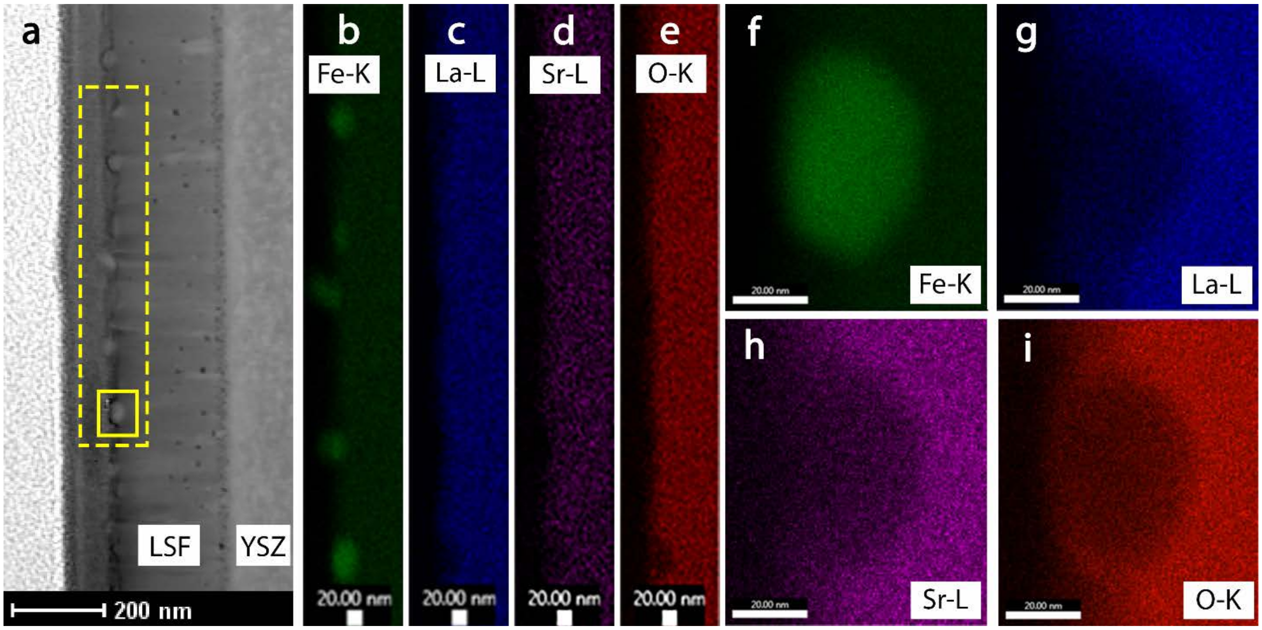

Supplementary Figure 8: Compositional analysis of the exsolved particles by energy dispersive X-ray spectroscopy. (a) High angle annular dark field image recorded in scanning transmission electron microscopy mode showing an overview of the regions for which elemental mapping was performed. (b)-(e) Mapping of the Fe, La, Sr, and O distribution in the region marked by the large dashed box in (a). The left part of the box contains the Pt-protection layer from focused ion beam preparation, which appears black in the elemental mapping. In (f)-(i) the elemental mapping of the particle within the small solid box in (a) is shown for Fe, La, Sr, and O.

Supplementary Tables

Supplementary Table 1: Fit results of the partial current-overpotential-curves from Figure 3

	left part (between ca. -400 mV and +150 mV) using Equation 10	right part (between ca. +150 mV and +400 mV) using Equation 11
I_0 / Acm^{-2}	$(1.03 \pm 0.04) \times 10^{-3}$	$(3.14 \pm 0.39) \times 10^{-4}$
β	0.5	0.29 ± 0.03

Supplementary References

1. Kabekkodu S. ICDD PDF-4+ Database. (International Centre for Diffraction Data) (2010).

2. Kuhn M, Hashimoto S, Sato K, Yashiro K, Mizusaki J. Oxygen Nonstoichiometry, Thermo-Chemical Stability and Lattice Expansion of $La_{0.6}Sr_{0.4}FeO_{3-\delta}$. *Solid State Ionics* **195**, 7-15 (2011).

3. Moreno R, Zapata J, Roqueta J, Bagués N, Santiso J. Chemical Strain and Oxidation-Reduction Kinetics of Epitaxial Thin Films of Mixed Ionic-Electronic Conducting Oxides Determined by X-Ray Diffraction. *J Electrochem Soc* **161**, F3046-F3051 (2014).

4. Schmid A, Rupp GM, Fleig J. Voltage and partial pressure dependent defect chemistry in $(La,Sr)FeO_{3-\delta}$ thin films investigated by chemical capacitance measurements. *Phys Chem Chem Phys* **20**, 12016-12026 (2018).

5. Nenning A, Opitz AK, Huber T, Fleig J. A Novel Approach for Analyzing Electrochemical Properties of Mixed Conducting Solid Oxide Fuel Cell Anode Materials by Impedance Spectroscopy. *Phys Chem Chem Phys* **16**, 22321-22336 (2014).

6. Baumann FS, Fleig J, Habermeier HU, Maier J. Impedance spectroscopic study on well-defined $(La,Sr)(Co,Fe)O_{3-\delta}$ model electrodes. *Solid State Ionics* **177**, 1071-1081 (2006).
